# Associations of plasma von Willebrand Factor levels with cognitive decline and neurodegeneration in older adults without dementia

**DOI:** 10.3389/fnagi.2025.1595071

**Published:** 2025-09-03

**Authors:** Pan Fu, Meiling Hu

**Affiliations:** Department of Neurology, Taizhou First People's Hospital, Taizhou, China

**Keywords:** Alzheimer’s disease, von Willebrand Factor, cognitive decline, structural MRI, brain atrophy

## Abstract

**Background:**

Previous studies have suggested that von Willebrand Factor (VWF) may be implicated in the pathogenesis of Alzheimer’s disease (AD). However, the association between plasma VWF levels and cognitive decline and neurodegeneration in older adults without dementia remains unclear.

**Methods:**

We investigated the cross-sectional and longitudinal associations between plasma von Willebrand Factor (VWF) levels and cognitive decline, as measured by the Mini-Mental State Examination (MMSE) and the Clinical Dementia Rating Scale Sum of Boxes (CDR-SB), as well as the volumes of six brain regions: the hippocampus, entorhinal cortex, middle temporal gyrus, fusiform gyrus, ventricles, and whole brain. Linear mixed-effects models were used to assess the association between plasma VWF levels and longitudinal changes in cognitive function and neuroimaging markers over time.

**Results:**

The study cohort consisted of 340 older adults without dementia at baseline. We observed that lower plasma VWF levels were associated with a faster rate of cognitive decline (MMSE: coefficient = 0.204, 95% CIs = [0.030, 0.378], *p*-value = 0.021; CDR-SB: coefficient = −0.268, 95% CIs = [−0.374, −0.163], *p*-value <0.001). Additionally, lower plasma VWF levels were linked to a more rapid reduction in the volumes of the hippocampus (coefficient = 0.016, 95% CIs = [0.004, 0.027], *p*-value = 0.009), entorhinal cortex (coefficient = 0.031, 95% CIs = [0.014, 0.048], *p*-value <0.001), and fusiform gyrus (coefficient = 0.047, 95% CIs = [0.008, 0.085], *p*-value = 0.017), as well as a faster enlargement of the ventricles (coefficient = −0.380, 95% CIs = [−0.558, −0.203], *p*-value <0.001). However, no significant relationships were observed between plasma VWF levels and changes in the volumes of the middle temporal gyrus or the whole brain (all *p*-values > 0.05).

**Conclusion:**

Our findings may contribute to the growing body of knowledge on the vascular contributions to cognitive function and may help identify potential biomarkers for the early detection and intervention of AD.

## Introduction

Alzheimer’s disease (AD), the most common form of dementia, is characterized by the accumulation of amyloid and tau proteins in the brain, cognitive decline, and neurodegeneration (Jack et al., 2010; Querfurth and LaFerla, 2010). Structural magnetic resonance imaging (MRI) is a non-invasive imaging technique that enables the examination of brain atrophy, a critical indicator of neurodegenerative diseases (Jack et al., 1992; Fox et al., 1999; Braskie and Thompson, 2014). It provides detailed measurements of brain volume, including the hippocampus and entorhinal cortex, regions that are particularly susceptible to atrophy in early AD (Devanand et al., 2007).

von Willebrand Factor (VWF), a large multimeric glycoprotein critical to hemostasis and vascular integrity, is synthesized by endothelial cells and megakaryocytes (Starke et al., 2011; Leebeek and Eikenboom, 2016). VWF has been associated with inflammation, endothelial dysfunction, and the pathogenesis of various neurological conditions (Rauch et al., 2013). Two meta-analyses suggested potential relationships between blood VWF levels and cognitive impairment or dementia, although results from individual investigations are conflicting (Quinn et al., 2011; Loures et al., 2019). Additionally, most of the included studies in the two meta-analyses were cross-sectional. Several longitudinal studies have explored the association, yielding inconsistent results (Carcaillon et al., 2009; Gallacher et al., 2010; Wolters et al., 2018; Wu et al., 2023). Three studies found no significant association (Carcaillon et al., 2009; Gallacher et al., 2010; Wu et al., 2023), while one suggested a potential short-term risk of dementia, but not a long-term one (Wolters et al., 2018). Therefore, the relationship between blood VWF levels and cognitive decline over time remains inconclusive, highlighting the need for further research in this area. With regard to the relationship between plasma VWF and brain volumes, a previous study utilized an inflammation composite score based on five plasma markers (including VWF) measured in midlife to investigate its association with late-life brain volumes (Walker et al., 2017). The authors reported that higher composite inflammation scores were linked to reduced brain volumes. However, the association between plasma VWF levels and brain atrophy remains less clear among older adults without dementia.

This study aimed to investigate the relationship between plasma VWF levels and cognitive decline, as well as its association with brain atrophy as measured by structural MRI among older adults. By clarifying these relationships, our study may contribute to the emerging body of knowledge on the vascular contributions to cognitive health and may help identify potential biomarkers for early detection and intervention for AD.

## Methods and materials

### Alzheimer’s disease neuroimaging initiative (ADNI)

Cross-sectional and longitudinal data used in the current study were extracted from the ADNI database (adni.loni.usc.edu). ADNI was launched in 2003 as a collaborative effort between the National Institute on Aging, the National Institute of Biomedical Imaging and Bioengineering, the Food and Drug Administration, private pharmaceutical companies, and non-profit organizations. Study participants have been enrolled from more than 50 centers across the USA and Canada. The ADNI study aims to investigate whether a combination of biomarkers, including neurocognitive assessments, neuroimaging, and fluid markers, can be used to track the progression of mild cognitive impairment (MCI) and early AD dementia (Petersen et al., 2010). This study received approval from the institutional review boards of all participating institutions, and written informed consent was provided by all study subjects or their authorized representatives.

### Sample participants

Our study sample included individuals with normal cognition (NC) and MCI (Petersen et al., 2010). Assignment to the NC group required a Mini-Mental State Examination (MMSE) (Folstein et al., 1975) score between 24 and 30 and a Clinical Dementia Rating (CDR) (Morris, 1993) score of 0. The diagnostic criteria for MCI included an MMSE score ranging from 24 to 30, a CDR of 0.5, the presence of a subjective memory complaint, evidence of objective memory loss as assessed by education-adjusted scores on the Wechsler Memory Scale Logical Memory II and essentially preserved ability to perform daily living activities. A total of 1821 participants with NC and MCI from ADNI1, ADNI-GO, ADNI2, and ADNI3 were initially considered for this analysis. From these, we selected 340 participants who met several criteria: availability of baseline plasma VWF data, at least two structural MRI scans, and demographic and clinical data at baseline, such as age, sex, education, and APOE4 genotype.

### Cognitive assessments

To examine the relationship between plasma VWF levels and cognitive decline over time, we used MMSE and Clinical Dementia Rating-Sum of Boxes (CDR-SB) (Williams et al., 2013) scores as cognitive outcomes. The MMSE is a measure of global cognition, with scores ranging from 0 to 30. Lower scores indicate greater cognitive impairment. The CDR-SB is an assessment of cognitive and functional capabilities, with scores ranging from 0 to 18. Higher scores indicate more severe cognitive impairment.

### Structural MRI neuroimaging markers

The imaging protocols for the ADNI are detailed on the official website^1^ and have been previously documented (Jack et al., 2008). The ADNI team at the University of California, San Francisco, utilized FreeSurfer image analysis software^2^ to process the T1-weighted sagittal 3D MPRAGE sequences obtained from MRI scans. From the ADNIMERGE dataset, we derived the volumes of several key brain regions, including the hippocampus, entorhinal cortex, fusiform gyrus, middle temporal gyrus, ventricles, and the whole brain. To normalize for variations in head size that could affect brain volume, we employed the following formula to calculate the adjusted volumes: Adjusted volumes = (raw regional brain volume / total intracranial volume) × 1,000. While there is no consensus on which normalization method should be preferred over another (Voevodskaya et al., 2014), the proportional approach was chosen because of its ease of application in practice and its intuitiveness.

### Measurement of plasma VWF levels

Plasma levels of VWF were measured using a multiplex immunoassay panel based on Luminex technology, with specific procedures for plasma collection and measurement detailed on the website^3^ and described previously (Hu et al., 2012). In summary, a subset of plasma samples from the ADNI cohort was analyzed for VWF and other plasma proteins through a 190-analyte multiplex immunoassay panel. This panel, developed on the Luminex xMAP platform by Rules-Based Medicine (RBM), encompasses a broad range of proteins. The plasma VWF concentrations were reported in ug/mL. Samples were analyzed in singlicate, whereas quality control (QC) samples were run in duplicate. The lower assay limit of the analyte (i.e., VWF) is 0.7, the least detectable dose was 2.8, the RBM low plasma range was 5.3, and the RBM high plasma range was 74. To evaluate the variability across distinct concentration ranges for the analyte, three levels of QC (low, medium, and high) were run, yielding coefficient of variation (CV) values of 28.8, 14.6, and 13.7%, respectively. For our statistical analysis, we employed quality-controlled plasma VWF values, which were then log-transformed.

### Statistical analysis

In cross-sectional analyses, descriptive statistics were performed to summarize the sample data. For example, mean (SD) was used to summarize continuous variables, and sample size (percentage) was used for categorical variables. Pearson’s correlation tests were conducted to investigate the correlations between plasma VWF levels and cognitive assessments, as well as structural MRI neuroimaging markers, among older adults without dementia. In longitudinal analyses, a total of eight linear mixed-effects models were built for eight different dependent variables, including cognitive measures (MMSE and CDR-SB) and neuroimaging markers (volumes of the hippocampus, entorhinal cortex, middle temporal gyrus, fusiform gyrus, ventricles, and whole brain). Each model adjusted for several potential covariates, including age, sex, education, and APOE4 status. For neuroimaging models, we additionally adjusted for baseline MMSE scores. Each model included the main effects of plasma VWF and potential covariates, as well as their interactions with time (follow-up duration in years). A random intercept for each participant was included in the model. All statistical analyses were conducted using R statistical software. We set the level of statistical significance at a two-tailed *p*-value of less than 0.05.

## Results

### Sample characteristics

Table 1 demonstrates the demographic characteristics and neuroimaging data of the study sample: 340 older adults without dementia, comprising 52 cognitively unimpaired participants and 288 MCI subjects. The mean age in the study sample was 74 years (SD = 7), and the mean educational level was 16 years of education (SD = 3). Females constituted 38% of the total participants, and APOE4 carriers accounted for half of the study sample. The mean adjusted volumes of the hippocampus, entorhinal cortex, middle temporal gyrus, fusiform gyrus, ventricles, and whole brain were 4.21 (SD = 0.73), 2.19 (SD = 0.49), 12.10 (SD = 1.62), 10.59 (SD = 1.39), 26 (SD = 13), and 643 (SD = 42), respectively. The mean plasma VWF levels were 1.64 (SD = 0.36) ug/mL. The comparison of variables between CU and MCI individuals is also listed in Table 1.

**Table 1:** Sample characteristics.

Characteristic	CU *N* = 52	MCI *N* = 288	*p*-value
Age, years	75 (6)	74 (7)	0.8
Education, years	16 (3)	16 (3)	0.7
Sex			0.11
Male	27 (52%)	183 (64%)	
Female	25 (48%)	105 (36%)	
APOE4 status			<0.001
APOE4 noncarriers	47 (90%)	122 (42%)	
APOE4 carriers	5 (9.6%)	166 (58%)	
MMSE	29 (1)	27 (2)	<0.001
CDR-SB	0.03 (0.12)	1.55 (0.82)	<0.001
Hippocampus	4.88 (0.62)	4.09 (0.68)	<0.001
Entorhinal cortex	2.58 (0.45)	2.13 (0.46)	<0.001
Middle temporal gyrus	13.03 (1.19)	11.94 (1.63)	<0.001
Fusiform gyrus	11.32 (1.38)	10.46 (1.35)	<0.001
Ventricles	20 (10)	27 (13)	<0.001
Whole brain	665 (42)	639 (41)	<0.001
Duration of follow-up, years	5.4 (4.2)	3.5 (2.7)	0.001
Annual change of MMSE	−0.06 (0.32)	−1.09 (1.04)	<0.001
Annual change of CDR-SB	−0.02 (0.10)	0.80 (0.73)	<0.001
Annual change of hippocampus	−0.05 (0.03)	−0.11 (0.05)	<0.001
Annual change of entorhinal cortex	−0.02 (0.03)	−0.06 (0.03)	<0.001
Annual change of middle Temporal gyrus	−0.10 (0.14)	−0.30 (0.20)	<0.001
Annual change of fusiform gyrus	−0.04 (0.12)	−0.18 (0.13)	<0.001
Annual change of ventricles	1.08 (0.53)	2.26 (1.27)	<0.001
Annual change of whole brain	−3.1 (3.6)	−6.9 (4.0)	<0.001
Conversion to dementia, *n* (%)	0 (0%)	130 (45%)	<0.001
Plasma VWF levels, ug/mL	1.66 (0.35)	1.64 (0.37)	0.8

Continuous variables and categorical variables were compared between two groups using Wilcoxon rank sum tests and Pearson’s Chi-squared tests, respectively. Brain volumes are adjusted volumes.

APOE4, Apolipoprotein E; MMSE, Mini-Mental State Examination; CDR-SB, Clinical Dementia Rating Sum of Boxes; NC, Normal Cognition; MCI, Mild Cognitive Impairment; VWF, von Willebrand Factor.

### Cross-sectional relationships between plasma VWF levels and cognitive performance and structural neuroimaging markers

**Figure 1:**
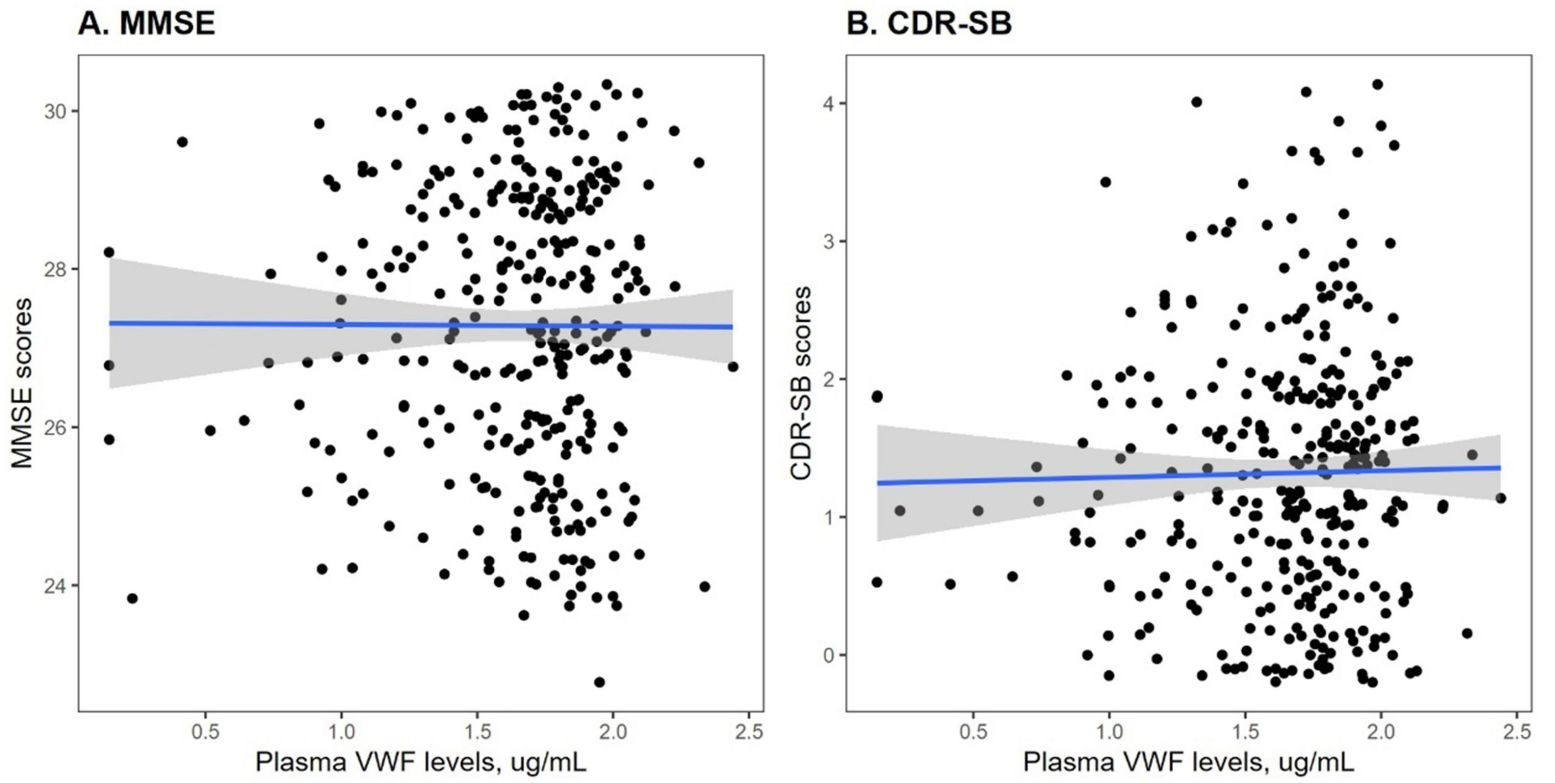
Correlation between plasma VWF levels and cognitive performance among older adults without dementia. Panel **A** shows the correlation between plasma VWF levels and MMSE scores. Panel **B** shows the correlation between plasma VWF levels and CDR-SB scores. MMSE: Mini-Mental State Examination; CDR-SB: Clinical Dementia Rating Sum of Boxes; VWF: von Willebrand Factor.

To understand the potential role of VWF in cognitive function, the correlations between plasma VWF levels and cognitive measures were analyzed in this study sample using Pearson correlation tests (Figure 1). As shown in Figure 1A, plasma VWF levels were not correlated with MMSE score among older adults without dementia (*r* = −0.004; *p* = 0.94). Similarly, as shown in Figure 1B, levels of plasma VWF were also not related with CDR-SB score (*r* = 0.019; *p* = 0.73).

**Figure 2:**
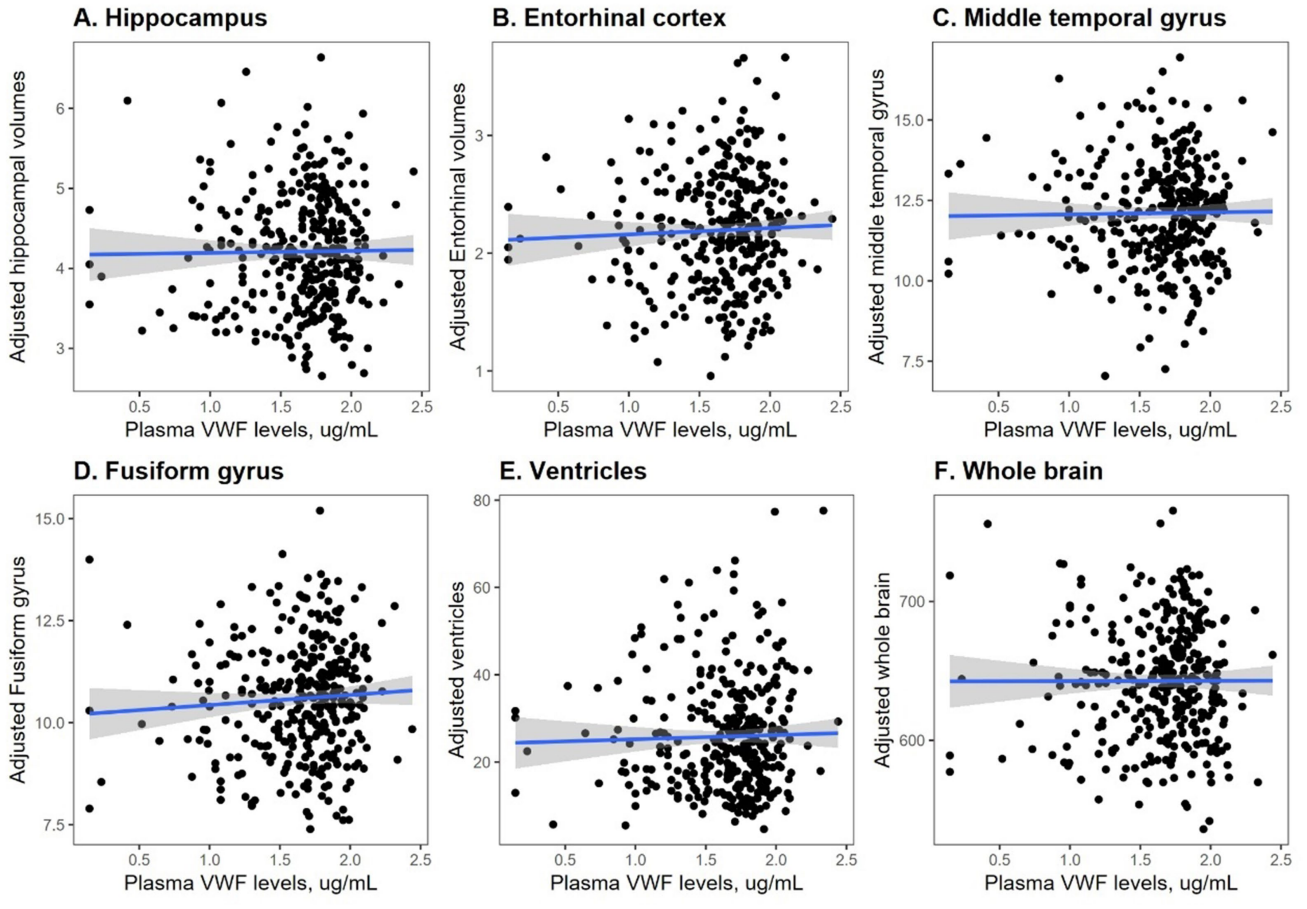
Correlation between plasma VWF levels and six structural MRI markers. Panel **A** shows the correlation between plasma VWF levels and the hippocampus. Panel **B** shows the correlation between plasma VWF levels and the entorhinal cortex. Panel **C** shows the correlation between plasma VWF levels and the middle temporal gyrus. Panel **D** shows the correlation between plasma VWF levels and the fusiform gyrus. Panel **E** shows the correlation between plasma VWF levels and the ventricles. Panel **F** shows the correlation between plasma VWF levels and the whole brain. VWF: von Willebrand Factor.

To understand the potential role of VWF in neurodegeneration, the correlations between plasma VWF levels and structural neuroimaging markers were analyzed in this study sample using Pearson correlation tests (Figure 2). As shown in Figure 2A, plasma VWF levels were not correlated with the volumes of the hippocampus among older adults without dementia (*r* = 0.01; *p* = 0.82). As shown in Figure 2B, levels of plasma VWF were also not related to the volumes of the entorhinal cortex (*r* = 0.04; *p* = 0.46). As shown in Figure 2C, levels of plasma VWF were also not related to the volumes of the middle temporal gyrus (*r* = 0.014; *p* = 0.8). As displayed in Figure 2D, levels of plasma VWF were not related to the volumes of fusiform gyrus (*r* = 0.06; *p* = 0.24). As demonstrated in Figure 2E, levels of plasma VWF were not related to the volumes of the ventricles (*r* = 0.03; *p* = 0.62). As displayed in Figure 2F, levels of plasma VWF were not related to the volumes of the whole brain (*r* = 0.002; *p* = 0.97).

Additionally, we further ran eight multivariable linear regression models for eight outcomes, adjusting for covariates. Model summaries are presented in Supplementary Tables S1, S2.

### Cross-sectional relationships between plasma VWF levels and CSF AD biomarkers

In a subset of 188 participants who had measurements for both plasma VWF and CSF AD biomarkers (i.e., Aβ42, total tau, and p-tau181 levels) at baseline, Pearson’s correlation tests were performed to examine the associations of plasma VWF levels with CSF Aβ42, total tau, and p-tau181 levels. No significant associations were found between plasma VWF levels and CSF Aβ42 (*r* = −0.036, *p* = 0.6), total tau (*r* = −0.008, *p* = 0.9), or p-tau181 (*r* = −0.02, *p* = 0.7) levels. Additionally, multivariable linear regression models were adjusted for several covariates, including age, education level, sex, and APOE4 status. The results remained unchanged. Plasma VWF levels were not significantly associated with CSF Aβ42 (coefficient = −86.2, SE = 78.9, *p* = 0.28), total tau (coefficient = −3.1, SE = 22, *p* = 0.89), or p-tau181 (coefficient = −0.7, SE = 2.5, *p* = 0.78) levels.

### Associations between plasma VWF levels and cognitive decline and the rates of brain atrophy

**Figure 3:**
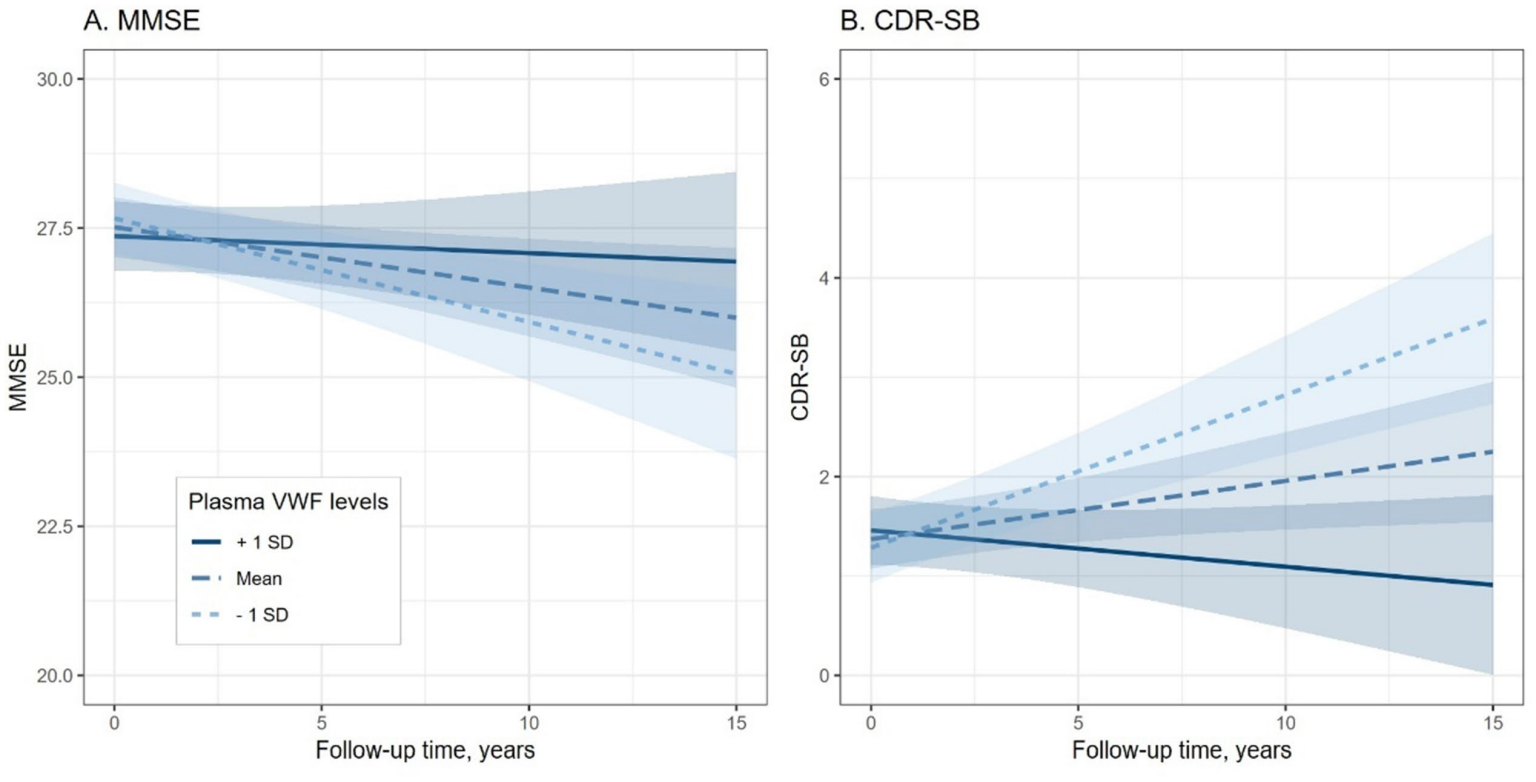
Association of plasma VWF levels and Cognitive decline among older adults without dementia. Panel **A** shows the association between plasma VWF levels and changes in MMSE scores. Panel **B** shows the association between plasma VWF levels and changes in CDR-SB scores. In the statistical model, plasma VWF levels were analyzed as a continuous variable. The stratification of values into three groups (1 SD above the mean, mean, and 1 SD below the mean) was employed for visualization purposes. MMSE: Mini-Mental State Examination; CDR-SB: Clinical Dementia Rating Sum of Boxes; VWF: von Willebrand Factor.

To explore the association between plasma VWF levels and longitudinal cognitive decline, the potential relationship between plasma VWF levels and changes in two cognitive measures was analyzed using linear mixed-effects models (Table 2). Plasma levels of VWF were significantly associated with changes in MMSE (lower scores represent worse cognitive performance; Coefficients [95% CIs] = 0.204 [0.030, 0.378]; *p* value = 0.021; Figure 3A) and CDR-SB (higher scores represent worse cognitive performance; Coefficients [95% CIs] = −0.268 [−0.374, −0.163]; *p* value < 0.001; Figure 3B) over time among older adults without dementia, suggesting that lower levels plasma VWF were associated with a steeper rate of cognitive decline. In addition, Supplementary Table S3 presents all coefficients of the main effects of the predictors.

**Table 2:** Linear mixed-effects models for cognitive measures.

Predictors	MMSE		CDR-SB	
	Estimates [95% CIs]	*p* values	Estimates [95% CIs]	*p* values
Age × Time	−0.005 [−0.014, 0.004]	0.263	0.007 [0.002, 0.013]	0.011
Female sex × Time	−0.254 [−0.377, −0.131]	<0.001	0.218 [0.144, 0.292]	<0.001
Education × Time	−0.017 [−0.034, 0.001]	0.069	0.017 [0.006, 0.028]	0.002
APOE4 carriers × Time	−0.639 [−0.754, −0.523]	<0.001	0.555 [0.485, 0.625]	<0.001
Plasma VWF × Time	0.204 [0.030, 0.378]	0.021	−0.268 [−0.374, −0.163]	<0.001

The models included the coefficients of the main effects of the predictors, but these coefficients were not shown in the table for the sake of brevity.

MMSE, Mini-Mental State Examination; CDR-SB, Clinical Dementia Rating Sum of Boxes; CIs, Confidence intervals; APOE4, Apolipoprotein E; VWF, von Willebrand Factor.

**Figure 4:**
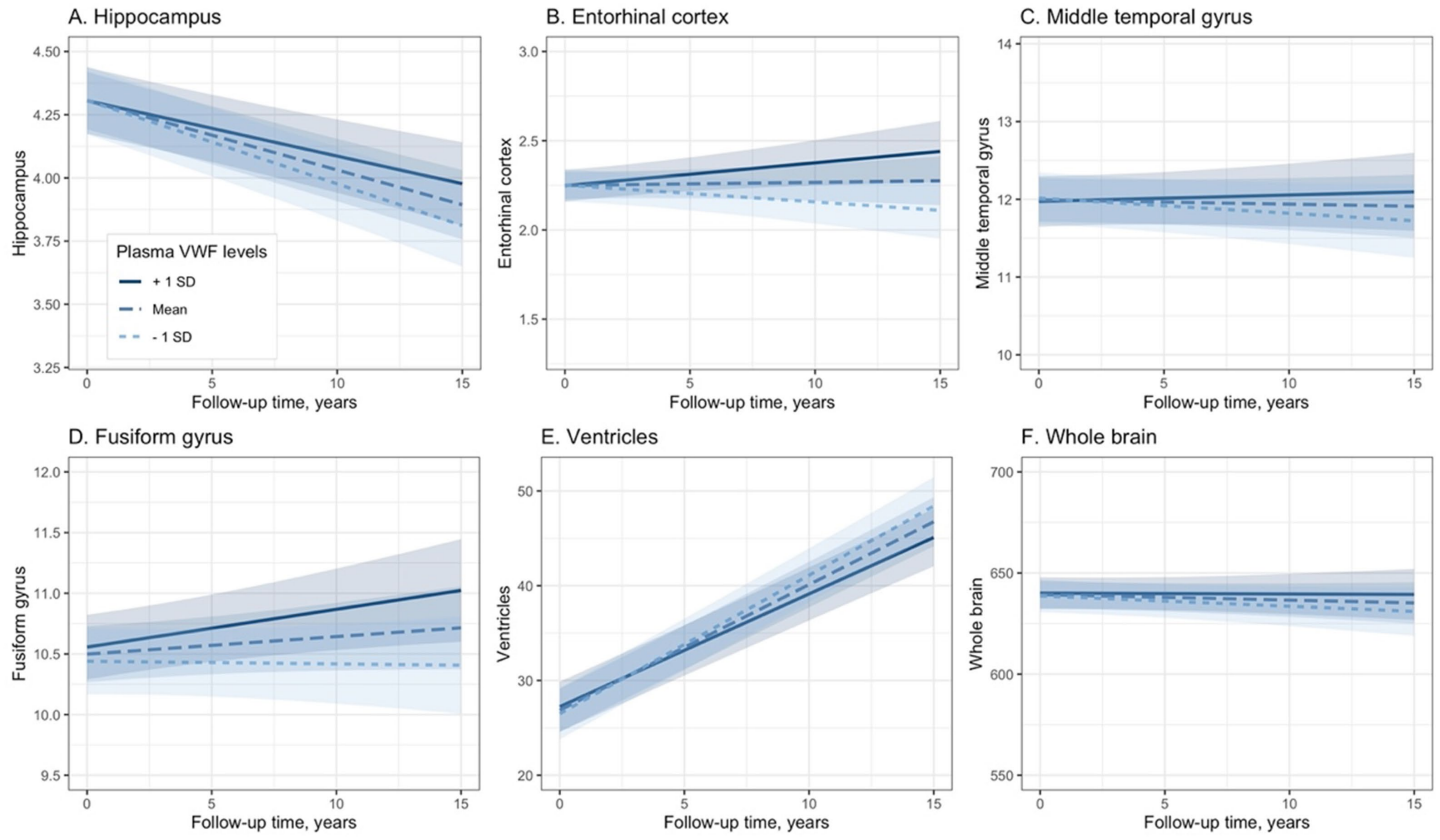
Association between plasma VWF levels and longitudinal changes in the six neuroimaging markers. Panel **A** shows the association between plasma VWF levels and the hippocampus. Panel **B** shows the association between plasma VWF levels and the entorhinal cortex. Panel **C** shows the association between plasma VWF levels and the middle temporal gyrus. Panel **D** shows the association between plasma VWF levels and the fusiform gyrus. Panel **E** shows the association between plasma VWF levels and the ventricles. Panel **F** shows the association between plasma VWF levels and the whole brain. In the statistical model, plasma VWF levels were analyzed as a continuous variable. The stratification of values into three groups (1 SD above the mean, mean, and 1 SD below the mean) was employed for visualization purposes. VWF: von Willebrand Factor.

To explore the association between plasma VWF levels and brain atrophy, the potential relationship between plasma VWF levels and changes in 6 structural neuroimaging markers was analyzed using linear mixed-effects models (Table 3). Plasma levels of VWF were significantly associated with changes in volumes of the hippocampus (Coefficients [95% CIs] = 0.016 [0.004, 0.027]; *p* value = 0.009; Figure 4A), entorhinal cortex (Coefficients [95% CIs] = 0.031 [0.014, 0.048]; *p* value < 0.001; Figure 4B), fusiform gyrus (Coefficients [95% CIs] = 0.047 [0.008, 0.085]; *p* value = 0.017; Figure 4D), and ventricles (Coefficients [95% CIs] = −0.380 [−0.558, −0.203]; *p* value < 0.001; Figure 4E) over time among older adults without dementia. In contrast, plasma levels of VWF were not associated with changes in volumes of the middle temporal gyrus (Coefficients [95% CIs] = 0.039 [−0.006, 0.083]; *p* value = 0.089; Figure 4C) or the whole brain (Coefficients [95% CIs] = 0.661 [−0.508, 1.830]; *p* value = 0.268; Figure 4F). In addition, Supplementary Table S4 presents all coefficients of the main effects of the predictors.

**Table 3:** Linear mixed-effects models for structural neuroimaging markers.

Predictors	Hippocampus		Entorhinal cortex		Middle temporal gyrus	
	Estimates [95% CIs]	*p* values	Estimates [95% CIs]	*p* values	Estimates [95% CIs]	*p* values
Age × Time	−0.001 [−0.002, −0.001]	<0.001	−0.001 [−0.002, −0.001]	0.002	−0.002 [−0.004, 0.000]	0.071
Female sex × Time	−0.038 [−0.047, −0.030]	<0.001	−0.038 [−0.050, −0.025]	<0.001	−0.122 [−0.154, −0.090]	<0.001
Education × Time	−0.003 [−0.004, −0.001]	<0.001	−0.005 [−0.007, −0.003]	<0.001	−0.013 [−0.017, −0.008]	<0.001
APOE4 carriers × Time	−0.066 [−0.074, −0.058]	<0.001	−0.041 [−0.053, −0.029]	<0.001	−0.208 [−0.239, −0.177]	<0.001
MMSE × Time	0.008 [0.005, 0.011]	<0.001	0.008 [0.005, 0.012]	<0.001	0.028 [0.018, 0.038]	<0.001
Plasma VWF × Time	0.016 [0.004, 0.027]	0.009	0.031 [0.014, 0.048]	<0.001	0.039 [−0.006, 0.083]	0.089

Predictors	Fusiform Gyrus		Ventricles		Whole brain	
	Estimates [95% CIs]	*p* values	Estimates [95% CIs]	*p* values	Estimates [95% CIs]	*p* values
Age × Time	−0.003 [−0.005, −0.001]	0.003	0.004 [−0.005, 0.014]	0.342	−0.044 [−0.105, 0.017]	0.154
Female sex × Time	−0.054 [−0.082, −0.027]	<0.001	0.271 [0.146, 0.396]	<0.001	−2.087 [−2.922, −1.251]	<0.001
Education × Time	−0.008 [−0.012, −0.004]	<0.001	0.030 [0.011, 0.049]	0.002	−0.218 [−0.343, −0.092]	<0.001
APOE4 carriers × Time	−0.151 [−0.178, −0.124]	<0.001	0.696 [0.571, 0.820]	<0.001	−4.254 [−5.079, −3.429]	<0.001
MMSE × Time	0.033 [0.024, 0.041]	<0.001	−0.137 [−0.177, −0.098]	<0.001	0.827 [0.564, 1.089]	<0.001
Plasma VWF × Time	0.047 [0.008, 0.085]	0.017	−0.380 [−0.558, −0.203]	<0.001	0.661 [−0.508, 1.830]	0.268

The models included the coefficients of the main effects of the predictors, but these coefficients were not shown in the table for the sake of brevity. Brain volumes are adjusted volumes.

CIs, Confidence intervals; MMSE, Mini-Mental State Examination; APOE4, Apolipoprotein E; VWF, von Willebrand Factor.

Additionally, for models with cognitive measures as outcomes, we further added cognitive status as a covariate. The results did not change (Supplementary Table S5).

## Discussion

We found that lower plasma VWF levels were associated with a faster rate of cognitive decline, as measured by the MMSE and the CDR-SB. Additionally, we found that lower plasma VWF levels were linked to a faster reduction in the volumes of the hippocampus, entorhinal cortex, and fusiform gyrus, as well as a faster enlargement of the ventricles. However, no significant relationships were observed between plasma VWF levels and changes in the volumes of the middle temporal gyrus or the whole brain. Our findings may contribute to the growing body of knowledge on the vascular contributions to cognitive abilities and may help identify potential biomarkers for early detection and intervention for AD.

Our results indicated that lower plasma VWF levels were associated with a faster rate of cognitive decline, as evidenced by scores on the MMSE and the CDR-SB. This finding is not consistent with previous studies. For example, two meta-analyses suggested potential relationships between higher blood VWF levels and an increased risk of vascular dementia, although results from individual investigations are conflicting (Quinn et al., 2011; Loures et al., 2019). Three studies found no significant association (Carcaillon et al., 2009; Gallacher et al., 2010; Wu et al., 2023), while one suggested a potential short-term risk of dementia, but not a long-term one (Wolters et al., 2018). For example, Wolters and colleagues found that higher VWF antigen levels were associated with an increased short-term (within 3 years of follow-up), but not long-term (after 4 years of follow-up), risk of dementia in a population-based study (Wolters et al., 2018). This discrepancy might be attributed to several factors, including differences in sample composition, methods for measuring blood VWF levels, and the statistical approaches used to examine the longitudinal association between VWF levels and cognitive decline or dementia risk. More importantly, the ADNI participants generally do not exhibit high vascular pathology burdens, as the ADNI cohort is primarily focused on AD rather than vascular dementia. For instance, individuals with multi-infarct dementia or a Hachinski Ischemia Scale score ≤ 4 at the screening visit were excluded from the study. This may also partly explain the observed inconsistency.

The observed link between VWF levels and the rate of reduction in hippocampal, entorhinal cortical, and fusiform gyrus volumes, as well as the rate of ventricular enlargement, provided further insight into the potential role of VWF in neurodegenerative processes. These regions are known to be among some of the earliest and most severely affected areas in AD, and their atrophy is closely associated with memory loss and cognitive decline (Jack et al., 1992; Devanand et al., 2007; Nestor et al., 2008; Mattsson et al., 2019). Interestingly, our study did not find significant relationships between plasma VWF levels and changes in the volumes of the middle temporal gyrus or the whole brain. This could suggest that the effects of VWF on brain atrophy may be region-specific, potentially reflecting the distinct vascular supply and functional roles of different brain regions. Our findings may contribute to the identification of potential biomarkers for the early detection and intervention of AD.

This study has several limitations. First, the observational nature of our data limits our ability to establish causality, and interventional or experimental studies are needed to confirm the directionality of the observed associations. Second, our sample size may limit the generalizability of our findings. Future studies with larger and more diverse cohorts will be crucial to validate our results and explore the potential therapeutic implications of modulating VWF levels. Third, no significant associations were found between plasma VWF levels and MMSE scores in cross-sectional analysis. This could be due to the narrow range of observed MMSE scores among older adults without dementia. Fourth, in future studies, it is also important to examine the role of ADAMTS13 (A Disintegrin and Metalloproteinase with a ThromboSpondin type 1 motif, member 13) activity, since the effects of VWF are reliant on the proteolytic activity of ADAMTS13. For instance, a prior study reported an association between elevated ADAMTS13 activity (which cleaves VWF multimers and reduces their functional activity) and increased diabetes risk (de Vries et al., 2017). Given that diabetes is a known risk factor for cognitive decline and dementia, this pathway may warrant further investigation. Also, Wolters and colleagues reported an association between low ADAMTS13 activity and increased risk of dementia (Wolters et al., 2018). Fifth, the ADNI participants are predominately white (93.5%), thus limiting the generalizability of the findings. Sixth, given that the ADNI cohort primarily targets AD, participants with substantial vascular pathology are underrepresented. For instance, those with multi-infarct dementia or a Hachinski Ischemia Scale score ≤ 4 were excluded. This may also limit the generalizability of the findings. Seventh, while baseline plasma VWF levels were associated with cognitive decline over time, no significant difference in plasma VWF levels was found between NC and MCI groups at baseline. This may be partly due to the relatively small sample size in the NC group (*n* = 52) compared to the MCI group (*n* = 288). Further studies are needed to investigate this inconsistency. Eighth, we did not find any associations between plasma VWF levels and the CSF AD biomarkers. This indicates that the relationship between VWF levels and neurodegenerative or cognitive impairment may involve non-AD pathways, such as well-established vascular pathway. Future studies are needed to elucidate the underlying mechanisms. Ninth, plasma VWF levels were measured in singlicate, which may increase variability. Future studies should validate these RBM-based assay results against established methods (e.g., enzyme-linked immunosorbent assay, ELISA).

In conclusion, despite inconsistencies with prior studies, the observed association between lower VWF levels and faster cognitive decline, as well as specific patterns of brain atrophy, highlights the potential role of VWF in cognitive function and neurodegeneration. Further studies are needed to replicate these findings, given the exploratory nature of the study.

## Data Availability

Publicly available datasets were analyzed in this study. This data can be found at: the Alzheimer’s Disease Neuroimaging Initiative (ADNI) database (adni.loni.usc.edu).

## Footnotes

^1^ https://adni.loni.usc.edu/data-samples/adni-data/neuroimaging/mri/

^2^ http://surfer.nmr.mgh.harvard.edu/

^3^ https://adni.loni.usc.edu/wp-content/uploads/2010/11/BC_Plasma_Proteomics_Data_Primer.pdf

## Group member of Alzheimer’s Disease Neuroimaging Initiative

Data used in the preparation of this article were obtained from the Alzheimer’s Disease Neuroimaging Initiative (ADNI) database (adni.loni.usc.edu). As such, the investigators within the ADNI contributed to the design and implementation of ADNI and/or provided data but did not participate in the analysis or writing of this report. A complete listing of ADNI investigators can be found at: http://adni.loni.usc.edu/wp-content/uploads/how_to_apply/ADNI_Acknowledgement_List.pdf.
