# Supplementary material for: Associations of plasma von Willebrand Factor levels with cognitive decline and neurodegeneration in older adults without dementia
Source: Front Aging Neurosci. 2025 Sep 3;17:1595071. doi: 10.3389/fnagi.2025.1595071 (PMC12440976; doi:10.3389/fnagi.2025.1595071)
Supplement: Supplementary file 1 [file Table_1.docx]

**Table S1: Summary of linear regression models with cognitive measures as dependent variables**

|  | **MMSE** | | **CDR-SB** | |
| --- | --- | --- | --- | --- |
| Predictors | Estimates [95% CIs] | P values | Estimates [95% CIs] | P values |
| (Intercept) | 27.722 [25.237, 30.208] | <0.001 | 2.027 [0.760, 3.294] | 0.002 |
| Age | -0.023 [-0.050, 0.004] | 0.099 | -0.011 [-0.025, 0.002] | 0.103 |
| Education | 0.097 [0.032, 0.161] | 0.003 | -0.013 [-0.046, 0.020] | 0.447 |
| Female sex | 0.008 [-0.392, 0.408] | 0.968 | -0.069 [-0.273, 0.135] | 0.508 |
| APOE4 carriers | -0.759 [-1.139, -0.379] | <0.001 | 0.455 [0.261, 0.648] | <0.001 |
| Plasma VWF | 0.071 [-0.458, 0.599] | 0.793 | 0.081 [-0.188, 0.350] | 0.554 |

|  | **Hippocampus** | | **Entorhinal Cortex** | | **Middle Temporal Gyrus** | |
| --- | --- | --- | --- | --- | --- | --- |
| Predictors | Estimates [95% CIs] | P values | Estimates [95% CIs] | P values | Estimates [95% CIs] | P values |
| (Intercept) | 6.884 [5.961, 7.807] | <0.001 | 2.843 [2.198, 3.488] | <0.001 | 14.921 [12.674, 17.168] | <0.001 |
| Age | -0.030 [-0.040, -0.020] | <0.001 | -0.010 [-0.017, -0.003] | 0.005 | -0.034 [-0.058, -0.010] | 0.006 |
| Education | -0.023 [-0.047, 0.001] | 0.061 | 0.007 [-0.010, 0.024] | 0.418 | -0.020 [-0.078, 0.038] | 0.498 |
| Female sex | 0.166 [0.018, 0.315] | 0.028 | 0.063 [-0.041, 0.167] | 0.235 | 0.134 [-0.227, 0.496] | 0.466 |
| APOE4 carriers | -0.425 [-0.566, -0.284] | <0.001 | -0.299 [-0.397, -0.200] | <0.001 | -0.365 [-0.708, -0.021] | 0.038 |
| Plasma VWF | 0.045 [-0.151, 0.242] | 0.649 | 0.066 [-0.072, 0.203] | 0.348 | 0.096 [-0.382, 0.573] | 0.694 |
|  | **Fusiform Gyrus** | | **Ventricles** | | **Whole brain** | |
|  | Estimates [95% CIs] | P values | Estimates [95% CIs] | P values | Estimates [95% CIs] | P values |
| (Intercept) | 13.761 [11.864, 15.659] | <0.001 | -19.530 [-36.350, -2.709] | 0.023 | 836.679 [784.067, 889.291] | <0.001 |
| Age | -0.045 [-0.065, -0.024] | <0.001 | 0.605 [0.424, 0.787] | <0.001 | -2.393 [-2.963, -1.824] | <0.001 |
| Education | -0.019 [-0.068, 0.031] | 0.458 | 0.081 [-0.354, 0.515] | 0.715 | -1.261 [-2.627, 0.104] | 0.070 |
| Female sex | 0.141 [-0.164, 0.446] | 0.365 | -5.221 [-7.914, -2.528] | <0.001 | 8.851 [0.386, 17.316] | 0.040 |
| APOE4 carriers | -0.229 [-0.519, 0.061] | 0.122 | 2.177 [-0.385, 4.739] | 0.096 | -11.149 [-19.189, -3.108] | 0.007 |
| Plasma VWF | 0.310 [-0.094, 0.713] | 0.132 | 0.070 [-3.493, 3.633] | 0.969 | 3.356 [-7.828, 14.540] | 0.555 |

**Table S2: Summary of linear regression models with brain volume measures as dependent variables**

**Table S3: Linear mixed-effects models for cognitive measures**

|  | **MMSE** | | **CDR-SB** | |
| --- | --- | --- | --- | --- |
| Predictors | Estimates [95% CIs] | P values | Estimates [95% CIs] | P values |
| Age | -0.010 [-0.054, 0.034] | 0.644 | -0.007 [-0.033, 0.019] | 0.617 |
| Years | 0.200 [-0.623, 1.024] | 0.633 | -0.291 [-0.794, 0.212] | 0.256 |
| Female sex | 0.019 [-0.631, 0.669] | 0.955 | -0.196 [-0.580, 0.188] | 0.317 |
| Education | 0.122 [0.018, 0.226] | 0.022 | -0.025 [-0.087, 0.036] | 0.422 |
| APOE4 carriers | -0.928 [-1.546, -0.311] | 0.003 | 0.380 [0.016, 0.745] | 0.041 |
| Plasma VWF | -0.420 [-1.282, 0.442] | 0.338 | 0.248 [-0.261, 0.757] | 0.339 |
| Age × Time | -0.005 [-0.014, 0.004] | 0.263 | 0.007 [0.002, 0.013] | 0.011 |
| Female × Time | -0.254 [-0.377, -0.131] | <0.001 | 0.218 [0.144, 0.292] | <0.001 |
| Education × Time | -0.017 [-0.034, 0.001] | 0.069 | 0.017 [0.006, 0.028] | 0.002 |
| APOE4 carriers × Time | -0.639 [-0.754, -0.523] | <0.001 | 0.555 [0.485, 0.625] | <0.001 |
| Plasma VWF × Time | 0.204 [0.030, 0.378] | 0.021 | -0.268 [-0.374, -0.163] | <0.001 |

**Table S4: Linear mixed-effects models for structural neuroimaging markers**

|  | **Hippocampus** | | **Entorhinal Cortex** | | **Middle Temporal Gyrus** | |
| --- | --- | --- | --- | --- | --- | --- |
| Predictors | Estimates [95% CIs] | P values | Estimates [95% CIs] | P values | Estimates [95% CIs] | P values |
| Age | -0.026 [-0.035, -0.016] | <0.001 | -0.008 [-0.015, -0.001] | 0.018 | -0.023 [-0.047, 0.001] | 0.066 |
| Years | -0.144 [-0.230, -0.058] | 0.001 | -0.099 [-0.224, 0.026] | 0.121 | -0.471 [-0.797, -0.146] | 0.005 |
| Female sex | 0.168 [0.022, 0.314] | 0.024 | 0.071 [-0.027, 0.169] | 0.154 | 0.177 [-0.183, 0.537] | 0.335 |
| Education | -0.030 [-0.054, -0.006] | 0.014 | 0.002 [-0.014, 0.018] | 0.819 | -0.036 [-0.095, 0.022] | 0.225 |
| APOE4 carriers | -0.338 [-0.479, -0.196] | <0.001 | -0.230 [-0.325, -0.135] | <0.001 | -0.131 [-0.481, 0.220] | 0.464 |
| MMSE | 0.105 [0.066, 0.144] | <0.001 | 0.062 [0.035, 0.088] | <0.001 | 0.248 [0.151, 0.345] | <0.001 |
| Plasma VWF | -0.002 [-0.194, 0.191] | 0.987 | -0.005 [-0.135, 0.125] | 0.941 | -0.058 [-0.535, 0.418] | 0.810 |
| Age × Time | -0.001 [-0.002, -0.001] | <0.001 | -0.001 [-0.002, -0.001] | 0.002 | -0.002 [-0.004, 0.000] | 0.071 |
| Female × Time | -0.038 [-0.047, -0.030] | <0.001 | -0.038 [-0.050, -0.025] | <0.001 | -0.122 [-0.154, -0.090] | <0.001 |
| Education × Time | -0.003 [-0.004, -0.001] | <0.001 | -0.005 [-0.007, -0.003] | <0.001 | -0.013 [-0.017, -0.008] | <0.001 |
| APOE4 carriers × Time | -0.066 [-0.074, -0.058] | <0.001 | -0.041 [-0.053, -0.029] | <0.001 | -0.208 [-0.239, -0.177] | <0.001 |
| MMSE × Time | 0.008 [0.005, 0.011] | <0.001 | 0.008 [0.005, 0.012] | <0.001 | 0.028 [0.018, 0.038] | <0.001 |
| Plasma VWF × Time | 0.016 [0.004, 0.027] | 0.009 | 0.031 [0.014, 0.048] | <0.001 | 0.039 [-0.006, 0.083] | 0.089 |
|  | **Fusiform Gyrus** | | **Ventricles** | | **Whole brain** | |
| Predictors | Estimates [95% CIs] | P values | Estimates [95% CIs] | P values | Estimates [95% CIs] | P values |
| Age | -0.032 [-0.052, -0.012] | 0.002 | 0.590 [0.392, 0.787] | <0.001 | -2.116 [-2.703, -1.528] | <0.001 |
| Years | -0.613 [-0.892, -0.334] | <0.001 | 4.923 [3.640, 6.206] | <0.001 | -17.388 [-25.915, -8.861] | <0.001 |
| Female sex | 0.124 [-0.171, 0.420] | 0.410 | -5.601 [-8.526, -2.676] | <0.001 | 10.281 [1.589, 18.973] | 0.021 |
| Education | -0.033 [-0.081, 0.016] | 0.185 | 0.185 [-0.293, 0.662] | 0.448 | -0.978 [-2.396, 0.441] | 0.176 |
| APOE4 carriers | -0.055 [-0.342, 0.233] | 0.708 | 1.837 [-1.005, 4.680] | 0.204 | -7.322 [-15.769, 1.125] | 0.089 |
| MMSE | 0.209 [0.129, 0.289] | <0.001 | -1.016 [-1.804, -0.229] | 0.012 | 3.117 [0.773, 5.461] | 0.009 |
| Plasma VWF | 0.163 [-0.227, 0.554] | 0.411 | 1.036 [-2.830, 4.902] | 0.598 | 1.889 [-9.605, 13.383] | 0.747 |
| Age × Time | -0.003 [-0.005, -0.001] | 0.003 | 0.004 [-0.005, 0.014] | 0.342 | -0.044 [-0.105, 0.017] | 0.154 |
| Female × Time | -0.054 [-0.082, -0.027] | <0.001 | 0.271 [0.146, 0.396] | <0.001 | -2.087 [-2.922, -1.251] | <0.001 |
| Education × Time | -0.008 [-0.012, -0.004] | <0.001 | 0.030 [0.011, 0.049] | 0.002 | -0.218 [-0.343, -0.092] | <0.001 |
| APOE4 carriers × Time | -0.151 [-0.178, -0.124] | <0.001 | 0.696 [0.571, 0.820] | <0.001 | -4.254 [-5.079, -3.429] | <0.001 |
| MMSE × Time | 0.033 [0.024, 0.041] | <0.001 | -0.137 [-0.177, -0.098] | <0.001 | 0.827 [0.564, 1.089] | <0.001 |
| Plasma VWF × Time | 0.047 [0.008, 0.085] | 0.017 | -0.380 [-0.558, -0.203] | <0.001 | 0.661 [-0.508, 1.830] | 0.268 |

**Table S5: Linear mixed-effects models for cognitive measures with cognitive status as an additional covariate**

|  | **MMSE** | | **CDR-SB** | |
| --- | --- | --- | --- | --- |
| Predictors | Estimates [95% CIs] | P values | Estimates [95% CIs] | P values |
| Age | -0.013 [-0.054, 0.029] | 0.551 | -0.004 [-0.028, 0.019] | 0.703 |
| Years | 0.384 [-0.419, 1.186] | 0.348 | -0.375 [-0.862, 0.112] | 0.131 |
| Female sex | -0.182 [-0.797, 0.433] | 0.561 | -0.041 [-0.385, 0.302] | 0.813 |
| Education | 0.113 [0.015, 0.211] | 0.024 | -0.018 [-0.073, 0.037] | 0.517 |
| APOE4 carriers | -0.511 [-1.131, 0.109] | 0.106 | 0.007 [-0.339, 0.353] | 0.968 |
| MCI diagnosis | -1.913 [-2.760, -1.066] | <0.001 | 1.666 [1.194, 2.137] | <0.001 |
| Plasma VWF | -0.580 [-1.394, 0.233] | 0.161 | 0.358 [-0.096, 0.812] | 0.122 |
| Age × Time | -0.007 [-0.015, 0.002] | 0.135 | 0.008 [0.003, 0.013] | 0.004 |
| Female × Time | -0.229 [-0.349, -0.109] | <0.001 | 0.199 [0.126, 0.271] | <0.001 |
| Education × Time | -0.015 [-0.033, 0.002] | 0.087 | 0.016 [0.006, 0.027] | 0.002 |
| APOE4 carriers × Time | -0.471 [-0.591, -0.351] | <0.001 | 0.448 [0.375, 0.520] | <0.001 |
| MCI diagnosis × Time | -0.476 [-0.598, -0.355] | <0.001 | 0.294 [0.221, 0.367] | <0.001 |
| Plasma VWF × Time | 0.293 [0.123, 0.464] | <0.001 | -0.331 [-0.433, -0.228] | <0.001 |
